# Protective role of *Codiaeum variegatum* against genotoxicity induced by carmustine in somatic and germ cells of male mice

**DOI:** 10.1007/s11033-022-07845-9

**Published:** 2022-09-02

**Authors:** Maha A. Fahmy, Ayman A. Farghaly, Entesar E. Hassan, Zeinab M. Hassan, Howaida I. Abd-Alla

**Affiliations:** 1 Department of Genetics and Cytology, National Research Centre, Giza, 12622 Egypt; 2 Chemistry of Natural Compounds Department, National Research Centre, Giza, 12622 Egypt

**Keywords:** Carmustine, Genotoxicity, Gene expression, *Codiaeum variegatum*, Protective role, HPLC-qTOF-MS/MS, *C*-glycosyl flavones

## Abstract

**Background:**

Carmustine (Cr) is an important chemotherapeutic drug, widely used in the treatment of brain tumors. Herein, the protective role of *Codiaeum variegatum* leaves ethyl acetate fraction was determined against genotoxicity of Cr. The technique HPLC-qTOF-MS/MS was used to identify the constituents in *C. variegatum*.

**Materials:**

90 male mice were used to evaluate micronuclei (MPCEs) in bone marrow, chromosomal aberration (CAs) in bone marrow and mouse spermatocytes, sperm abnormalities, and gene expression (*q*RT-PCR). The following groups were included, I: Negative control (ethanol 30%), II: Positive control (i.p injected once with 30 mg/kg Cr), III: Control orally treated with *C. variegatum* at 500 mg/kg, four days. IV-VI: treated with 100, 300, and 500 mg/kg of the plant (4 days) plus a single dose of Cr.

**Results:**

In bone marrow, Cr induced significant increase in MPCEs and CAs by 3 and 7-folds respectively over the control. Cr also induced a significant percentage of CAs in spermatocytes in meiosis in the form of univalent (X–Y and autosomal univalent) and also a significant percentage of morphological sperm abnormalities was recorded. A large number of coiled tail abnormalities were detected indicating the effect of Cr in sperm motility. Cr induced an overexpression of p53 gene. *C. variegatum* mitigated all deleterious genotoxic effects of Cr. Chemical analysis showed that flavones (35.21%) and phenolic acids (17.62%) constitute the main components.

**Conclusions:**

The results indicated that Cr is genotoxic in both somatic and germ cells. The active components in *C. variegatum* together participate in the obtained protective role.

## Introduction

*Codiaeum* is an important member of the family Euphorbiaceae, which includes about 300 genera and 8900 species, several of which are widely used in traditional medicine [1, 2]. Among them, *Codiaeum* is a valuable genus in the family and it includes approximately 200 species. Different *Codiaeum* species are used in folklore medicine. This genus was demonstrated to be rich in various bioactive components including mainly polyphenols [2, 3]. Phytochemicals, especially polyphenols are known to be responsible for antioxidant and free radical scavenging activities which are involved in many diseases such as cancer, diabetes, aging and cardiovascular problems [4]. *Codiaeum variegatum* was known as "garden croton῍ and has the common name Croton. Its leaves are considered with sedative, purgative, anti-amoebic, antioxidant activities, anti-inflammatory, and anticancerous properties [2]. Root decoction is taken to treat gastric ulcers. The fresh latex and leaves extract of *C. variegatum* are active against influenza A virus and herpes simplex virus, respectively [2]. Antifungal, antibacterial, insecticidal, antimalarial activity and treatment of kidney stones, and immunostimulant properties were also reported [2].

Carmustine (1,3-*bis*(2-chloroethyl)-1-nitrosourea) is an alkylating agent in the haloethylnitrosourea family and is also known by its brand name, BCNU. It is a common synthetic antineoplastic agent frequently employed, as a systemic monotherapy or in established combination therapy with other approved chemotherapeutic drugs or with other therapeutic measures (surgery, radiotherapy). It is used in the managements of various types of malignancies, *e.g.* brain tumors, multiple myeloma, Hodgkin’s and non-Hodgkin’s lymphomas [5]. It is highly lipophilic, capable of readily crossing the blood–brain barrier and thus it is useful and important as a chemotherapeutic agent against brain tumors [6, 7]. As with other nitrosoureas and under physiological conditions, it undergoes spontaneous nonenzymatic decomposition to release 2 reactive metabolites with alkylating and carbamoylating activities. Such metabolites are thought to be responsible for the antineoplastic activities of Cr [8]. It has been established as genotoxic both in vivo and in vitro and is classified as an animal carcinogen [9]. The most common adverse effects of Cr are bone marrow suppression, pulmonary toxicity, genotoxicity and fetal harm when administered to a pregnant woman [10, 11, 12]. It alkylates DNA and RNA and induces DNA cross-linking [7, 13]. In addition, secondary tumors were reported to be associated with the long-term use of nitrosoureas [7]. Recently, genotoxicity has fully developed into a serious question for the cause of several cancers. Due to the low toxicity, effective pharmacological activities and economic viability, new therapeutic applications of plants have been examined in the light of recent systematic developments throughout the world [14]. Accordingly, we decided to determine the genotoxic effect of carmustine at different cytogenetic endpoints and the possible ameliorative effect of different doses of the *C. variegatum* ethyl acetate fraction from leaves was evaluated. The main groups of metabolites and their active components in *C. variegatum* L. was demonstrated by high-performance liquid chromatography-quadrupole, time-of-flight, and tandem mass spectrometry (the data is under publications).

## Materials and methods

### Plant material

*Codiaeum variegatum* L. cv. Gold Dust leaves were collected from Al- Zohriya Garden, Giza, Egypt in 2019. The plant was kindly identified by Therese Labib, Herbarium Section, El-Orman Botanical Garden, Giza (Egypt). A voucher specimen (02Cva/2019) was prepared and deposited at the Chemistry of Natural Compounds Department, National Research Centre (NRC), Dokki-Giza (Egypt).

### Preparation of extract

The air-dried leaves of *C. variegatum* (1.8 kg) were crushed and extracted with 80℅ ethanol by maceration at room temperature. A rotary evaporator was used to evaporate the solvent under vacuum at 50 °C. The resulting ethanol extract was sequentially defatted and desalted by CHCl_3_ and C_2_H_5_OH, respectively, by warming under reflux conditions. Thereafter, the residue was taken in ethyl acetate affording an ethyl acetate-soluble fraction that yielded 14.96 g.

### Cytogenetic studies

#### Chemicals

Carmustine (Cr) was purchased from Sigma–Aldrich Corp. (St. Louis, MO,

USA), dissolved in ethanol 30% and injected i.p. in a single dose of 30 mg/kg body weight. This dose was comparable to the human therapeutic dose for the treatment of brain tumors [15] based on body surface area conversion ratios [16].

#### Experimental animals

Mature Swiss male mice of 8–12 weeks and weighing approximately 25 g were obtained from the animal house colony of the NRC, Dokki-Giza (Egypt). Mice were housed in plastic boxes in an air-conditioned room of temperature (23 ± 1 °C) and relative humidity (50 ± 20%) with a 12-h light/dark cycle. Mice were provided with a standard balanced pelleted chow diet and chlorinated tap water ad libitum.

#### Experimental design

In these experiments, a total of 90 mice were divided as follows: 30 mice for micronucleus test and the real-time polymerase chain reaction (real-time PCR), 30 mice for chromosomal aberrations in the bone marrow and mouse spermatocytes, and 30 mice for sperm abnormalities. In each of these tests, mice were subdivided into groups (5 /group).

The main groups in all tests examined were, **Group I**: Negative control (30% ethanol), **Group II**: Mice i.p injected with a single dose of Cr (positive control) at the dose level of 30 mg/kg, **Group III**: Plant control orally administrated the extract of *C. variegatum* at the highest tested dose (500 mg/kg) for 4 successive days, **Groups IV-VI:** Represent the combined groups in which mice received repeated oral treatment with *C. variegatum* fraction (100, 300 and 500 mg/kg) for four days and at the last day received i.p injection with Cr (30 mg/kg). In all experiments, except sperm analysis, samples were taken 24 h after the last treatment. In the test of sperm abnormalities, samples were taken 35 days after the 1^st^ injection.

### Cytogenetic analysis

#### Micronucleus test

The micronucleus preparation from bone marrow was performed following the standard test protocol of Schmid [17] and according to the Guideline OECD 474 for the Testing of Chemicals [18]. Briefly, the bone marrow cells were collected from bilateral femurs after separation in 3 mL of fetal bovine serum, centrifuged and smeared on slides. The air-dried slides were fixed by submerging in absolute methanol (for 10–20 min). Fixed slides were stained with May Grünwald—Giemsa protocol. Micronuclei were identified as dark blue staining bodies in the cytoplasm of polychromatic erythrocytes (MPCEs). The ratio of erythrocytes to nucleated cells was expressed as the percentage of PCE’s/100 nucleated cells (PCE᾽s + NCE᾽s). Total of 2000 cells (PCEs + NCEs) were scored/animal (5 animals/group). Scoring was performed under 1000 × magnification with a light microscope.

#### Chromosomal aberration assay in mouse bone marrow and spermatocytes

Mitotic and meiotic chromosomes were prepared from bone marrow and testis of the same animal, respectively. Bone marrow chromosomes were prepared according to the technique described by Fahmy et al. [19]. In brief, mouse bone-marrow cells were collected from both femurs, cells were incubated in hypotonic solution (KCL 0.075 M) for 20 min at 37 °C, and then centrifuged. The cell pellets were suspended in a fixative (methanol/glacial acetic acid 3:1). This step was repeated at least twice, then the cells were suspended in a few drops of fixative and spread onto frozen slides, air-dried, stained with 10% Giemsa for 30 min, washed, and air dried again.

Spermatocyte chromosomes were prepared from the testes according to the protocol described by Evans et al. [20] with some modifications [21]. Briefly, the testis was removed and squashed into a petri dish containing an isotonic solution of 2.2% trisodium citrate. Then the cell suspension was centrifuged for 5 min at 1500 rpm. The cell pellet was incubated in a hypotonic solution of 1.1% trisodium citrate for 20 min at 37 °C followed by centrifugation. The cell pellet was washed twice by a freshly prepared fixative. A few drops of the fixative cell suspension were dropped in clean microscopic slides, air-dried and stained with 10% Giemsa stain. In each, one hundred well-spread metaphases were analyzed per mouse describing different kinds of chromosome abnormalities (CAs). Scoring for CAs was performed under ×2000 magnification with a light microscope.

#### Morphological sperm abnormalities

Sperm were prepared according to the recommended method of Wyrobek and Bruce [22] with some modifications recorded by Fahmy et al. [19]. Smears were stained with 1% Eosin Y. A total of 1000 sperm was counted per animal (5000/each treatment), and different types of sperm abnormalities were scored (Head and Tail abnormalities). Sperm preparations were examined by light microscopy at ×1000 magnification.

### Expression of p53

#### Isolation of total RNA

Total RNA was isolated from liver tissues of male mice by the standard TRIzol® Reagent extraction method (cat#15596-026, Invitrogen, Germany). Briefly, tissue samples were homogenized in mortar with liquid nitrogen and 1 mL of TRIzol® Reagent. RNA was dissolved in diethylpyrocarbonate (DEPC)-treated water up to use.

#### Reverse transcription (RT) reaction

The complete Poly(A)^+^ RNA isolated from liver tissue samples was reverse transcribed into cDNA in a total volume of 20 µl using RevertAid™ First Strand cDNA Synthesis Kit (MBI Fermentas, Germany). 

#### Real time-polymerase chain reaction (qRT-PCR)

Step One™ Real-Time PCR System from Applied Biosystems (Thermo Fisher Scientific, Waltham, MA USA) was used to determine the tissues samples of mice copy number. The sequences of specific primer of the genes used are listed in Table 1. At the end of each qPCR a melting curve analysis was performed at 95.0 °C to check the quality of the used primers. The relative quantification of the target to the reference was determined by using the 2^*−*ΔΔCT^ method.

**Table 1:** Primers sequence used for *qRT-PCR*

Gene	Primers sequence	NCBI Reference
p53	F: ACA GTC GGA TAT CAG CCT CG	AB021961.1
	R: GCT TCA CTT GGG CCT TCA AA	
GAPDH	F:GGA TGC AGG GAT GAT GTT CT	NM_017008.3
	R:GAA GGG CTC ATT GAC CAC AGTT	

GAPDH: glyceraldehyde-3-phosphate dehydrogenase,

P53: tumor protein p53 gene

### Data analysis

Data were analyzed using computerized software SPSS (Statistical Package of Social Science, version 20, Armonk, New York: IBM Corp). The data were checked for normality and the homogeneity of the variance using the Kolmogorov–Smirnov's test and Levene's test, respectively. The differences among groups with normal distribution were analyzed by one-way analysis of variance (ANOVA) followed by the Tukey HSD test. The results were regarded as significant when the P-value was less than or equal to 0.05.

## Results

### Chemical composition of *C. variegatum*

Depending on the structural information obtained from HPLC/QTOF–MS/MS data, in [M + H]^−^ ion, total ion current chromatogram (TIC), a total of 115 components were unambiguously identified. The flavonoids were the main component identified (including flavones (35.21%) of *C*- and/or *O*-hexosides nature, flavonols (3.40%), isoflavones (0.53%). Also, the study can identify other important classes of chemicals *e.g.* phenolic acids (17.62%), coumarins (8.29%), indoles (4.10%), fatty acids (0.932%), amino acid derivatives (0.53%), and polyols (0.49%) based on their fragments (data of chemical analysis is under publication).

### Cytogenetic analysis

#### Micronucleus analysis

Table 2 presents the frequency of MPCEs induced after treatment of Cr with and without *C. variegatum* fraction at different doses (100,300 and 500 mg/kg). Also, the percentage of PCEs to the total counted cells was recorded. The results demonstrated a highly significant percentage of MPCEs after Cr treatment reached 9.88 ± 0.58 compared to 2.63 ± 0.63 and 2.97 ± 0.27 for negative and plant control respectively. The extract of *C. variegatum* at the dose levels of 300 and 500 mg/kg was significantly reduced such effect to be reached to 6.67 ± 0.49 and 5.75 ± 0.26 respectively. The results also indicated that Cr induced an increase in the number of PCEs/total counted cells, indicating bone marrow toxicity. The extract of *C. variegatum* at different doses ameliorates such percentage in a dose-dependent manner.

**Table 2:** Percentage of polychromatic erythrocytes (PCEs) and PCEs with micronuclei (MPCEs); chromosomal aberrations induced in mouse bone marrow cells after treatment with carmustine and *Codiaeum variegatum*

Treatment and doses	No. and percentage of PCEs/(PCEs + NCPs)		No. and percentage of MPCEs		Total abnormal metaphases		No and (%) of metaphases with different types of chromosome aberrations			
	NO	Mean ± S.E	NO	Mean ± S.E	No	Mean (%) ± SE	Gap	Fragment and/or break	Multiple aberrations	R.T
I. Control (30% ethanol)	419	4.19 ± 0.37^a^	11	2.63 ± 0.63^a^	23	4.60 ± 0.4 ^a^	7 (1.40)	16 (3.20)	–	–
II. Carmustine (30 mg / kg)	1448	14.48 ± 0.49^d^	143	9.88 ± 0.58^c^	163	32.60 ±0.0.75 ^e^	12 (2.40)	42 (8.40)	102 (20.40)	7 (1.40)
III. *Codiaeum variegatum* (500 mg/kg)	438	4.38 ± 0.54^a^	13	2.97 ± 0.27^a^	16	3.20 ± 0.58^a^	7 (1.40)	9 (1.80)	–	–
IV-VI- Carmustine + *Codiaeum variegatum*										
+ 100 mg/kg	1132	11.32 ± 0.53^c^	88	7.77 ± 0.61 ^c^	130	26.0 ± 0.89^d^	14 (2.80)	37 (7.40)	72 (14.40)	7 (1.40)
+ 300 mg/kg	1005	10.05 ± 0.49^b^	67	6.67 ± 0.49 ^b^	107	21.40 ± 0.98 ^c^	13 (2.60)	41 (8.20)	49 (9.80)	4 (0.80)
+ 500 mg/kg	887	8.87 ± 0.32^b^	51	5.75 ± 0.26 ^b^	86	17.20 ± 0.86^b^	11 (2.20)	35 (7.0)	36 (7.20)	4 (0.80)

A total of 500 cells were analyzed (5 mice per group; 100 cells/mouse) for chromosomal aberrations. R.T.: Robertsonian translocation. No. of examined nucleated cells = 2000/mouse (5 mice/group) for micronuclei test.One way ANOVA–Tukey’s multiple comparisons test was used. The values having different superscript letters in each column are significantly different from one another at *p* < 0.05

#### Chromosomal aberrations (CAs) in bone marrow cells

**Fig. 1:**
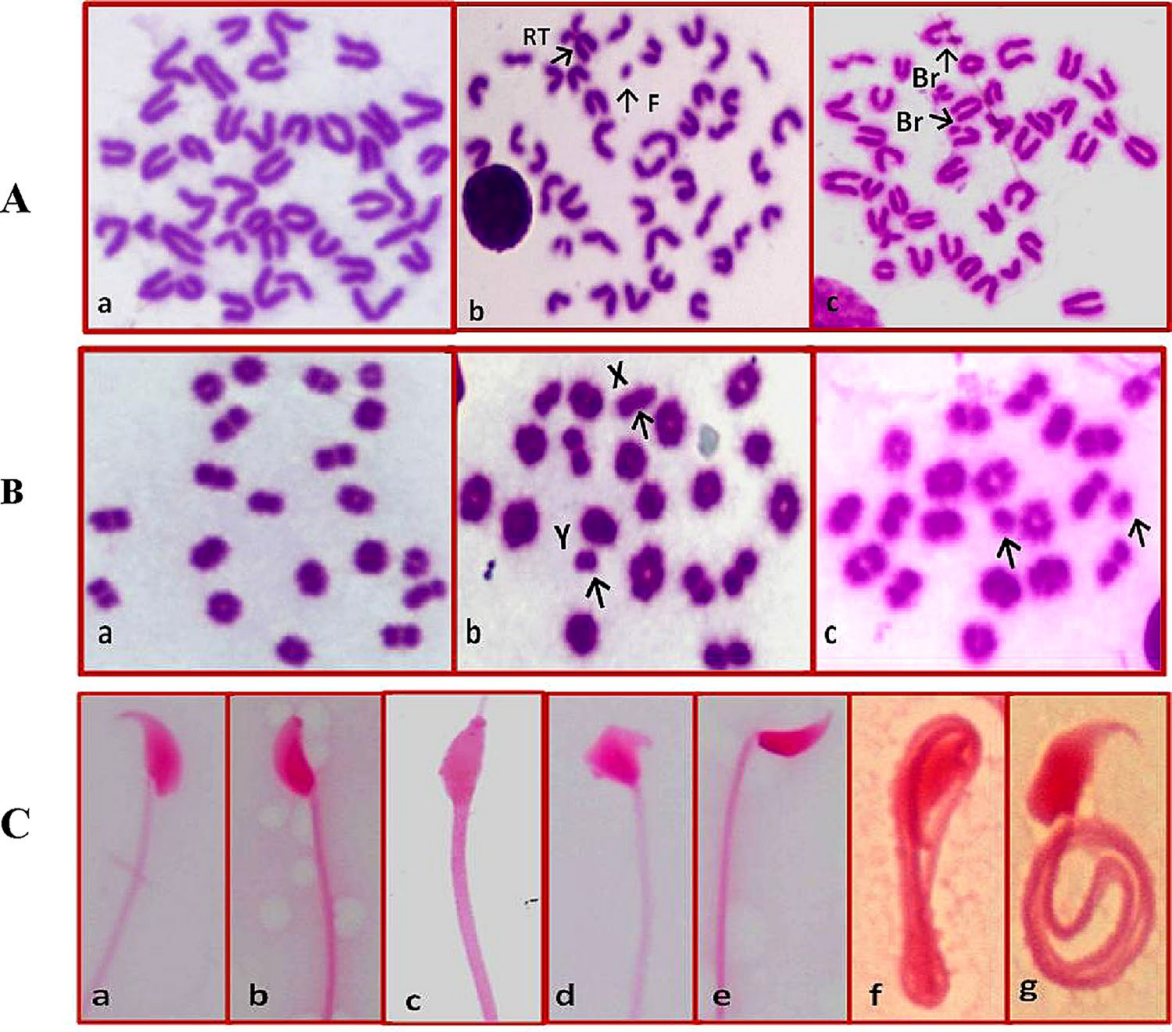
**A** Chromosomal abnormalities in bone marrow cells in mice showing (a) normal (b) fragment and Robertsonian translocation and (c) breaks. **B** Chromosomal abnormalities in diakinase- metephase 1 cells in mice showing (a) normal, (b) XY univalent and (c) autosomal univalent. **C** Sperm abnormalities in mice showing (a) normal, (b) without hook, (c) amorphous, (d) triangular, (e) banana and (f, g) coiled tail

Table 2 also revealed that Cr (30 mg/kg) induced a significant percentage of CAs in bone marrow cells reaching 32.60 ± 0.75 *vs* 4.60 ± 0.40 for control. Metaphases contain multiple aberrations and metaphases with breaks/fragments are the most pronounced after Cr treatment. Robertsonian translocations were also recorded (Fig. 1A). The results also demonstrated that *C. variegatum* at the highest tested dose (500 mg/kg) has approximately the same effect as the control negative. Moreover, the tested concentrations of *C. variegatum* significantly decreased Cr-induced aberrations recording a dose-dependent relationship.

#### Chromosomal aberrations in spermatocyte cells

Table 3 recorded that Cr induced a significant percentage of chromosomal aberrations in mouse spermatocytes reached 16.80 ± 0.97 *vs* 3.80 ± 0.37 and 3.20 ± 0.49 for control negative and control plant respectively. The three tested concentrations of *C. variegatum* are significantly mitigated the frequency of aberrations in a dose-dependent manner. The majority of aberrations induced after Cr treatment are univalent (X–Y and autosomal univalent (Fig. 1B).

**Table 3:** Percentage of chromosomal aberrations in mouse spermatocytes and sperm abnormalities induced after treatment with carmustine and *Codiaeum variegatum*

Treatment and doses	Total abnormal metaphases		No. of different types of sperm abnormalities		Total abnormal sperm	
	No	Mean(%) ± SE	Head abnormalities	Tail abnormalities	No	Mean (%) ± SE
I. Control (30% ethanol)	19	3.80 ± 0.37 ^a^	126	86	212	4.24 ± 0.19^a^
II.Carmustine (30 mg / kg)	84	16.80 ± 0.97^c^	274	252	526	10.52 ± 0.21^e^
III. *Codiaeum variegatum* (500 mg/kg)	16	3.20 ± 0.49 ^a^	122	104	226	4.52 ± 0.38^a^
IV-VI- Carmustine + *Codiaeum variegatum*						
+ 100 mg/kg	54	10.80 ± 0.97 ^b^	276	201	477	9.54 ± 0.46^d^
+ 300 mg/kg	46	9.20 ± 0.58^b^	191	191	382	7.64 ± 0.31^c^
+ 500 mg/kg	43	8.60 ± 0.75 ^b^	156	164	320	6.40 ± 0.24 ^b^

A total of 500 cells were analyzed for chromosomal aberrations (5 mice per group; 100 cells/mouse). Total number of examined sperms 5000 per each treatment (1000 /mouse, 5 mice/group). One way ANOVA–Tukey’s multiple comparisons test was used. The values having different superscript letters in each column are significantly different from one another at *p* < 0.05

#### Morphological sperm abnormalities

Sperm abnormalities induced after treatment with Cr and *C. variegatum* was presented in Table 3. The results indicated that Cr induced a significant percentage of sperm defects that reached 10.52 ± 0.21 *vs* 4.24 ± 0.19 for the negative control. The results also indicated that *C. variegatum* has approximately the same effect as control. Cr induced both head and tail abnormalities but the coiled tail abnormality was the most pronounced type of sperm defects induced after Cr treatment. Different doses of *C. variegatum* ameliorated such effect in a dose dependent manner. The maximum protective effect was recorded with the dose 500 mg/kg of plant extract. Figure 1C represents different types of the induced sperm abnormalities.

#### Gene expression of p53 gene as indicated by qRT-PCR

**Fig. 2:**
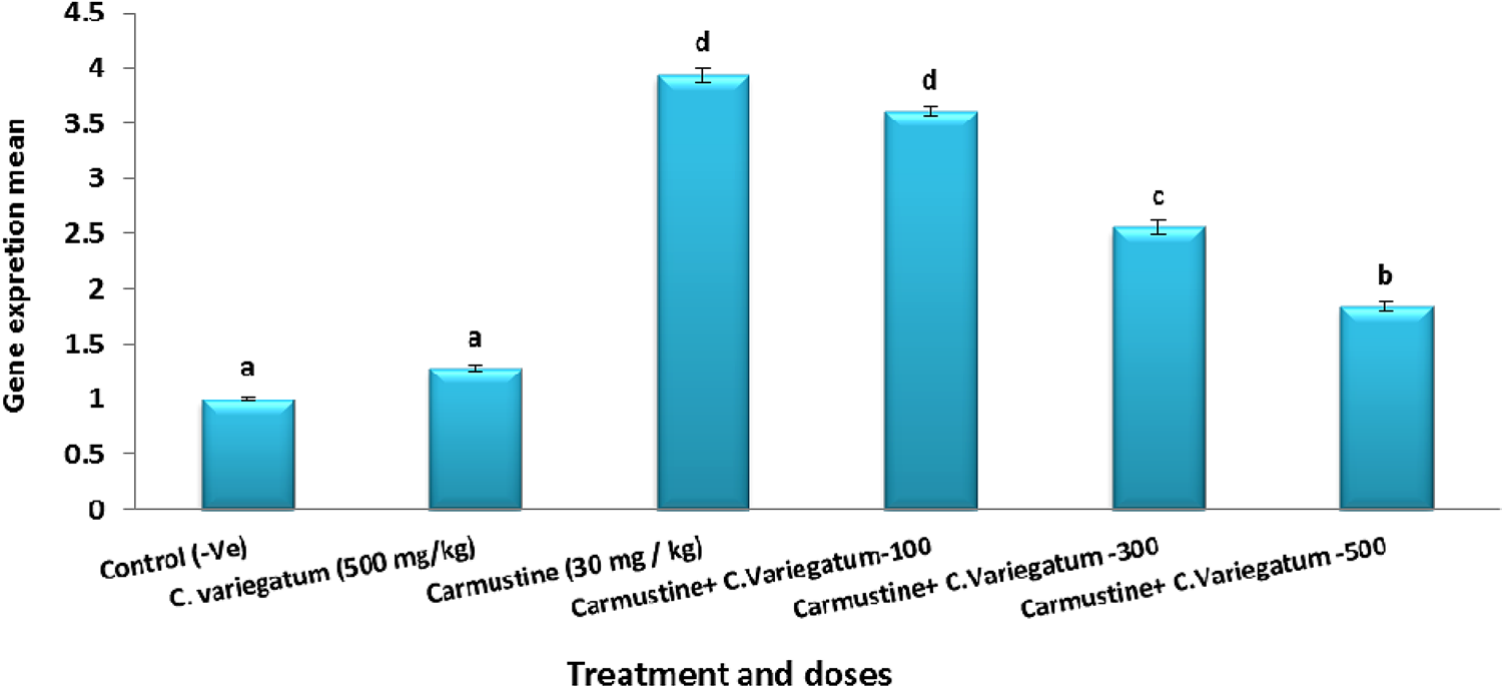
P53 gene expression as indicated by *q* RT-PCR in mice liver cells

The results in Fig. 2 and Table 4 indicated an over expression of the gene p53 after treatment with Cr 3.94 ± 0.07 *vs* 1.0 ± 0.02 and 1.28 ± 0.03 for the negative control and *Codiaeum variegatum* (500 mg/kg, control plant) respectively. The results also showed that the combined groups (Cr plus *C. variegatum*) have lower values of p53 expression than Cr group. The maximum modulating effect was demonstrated with the highest tested dose of plant where the value of p53 expression reached 1.84 ± 0.04.

**Table 4:** The gene expression of P53 gene as indicated by RT-PCR

Treatment	Mean	SEM
I. Control (30% ethanol)	1	0.02
II.Carmustine (30 mg / kg)	3.94	0.07
III. *Codiaeum variegatum* (500 mg/kg)	1.28	0.03
IV-VI- Carmustine + *Codiaeum variegatum*		
+ 100 mg/kg	3.61	0.04
+ 300 mg/kg	2.56	0.07
+ 500 mg/kg	1.84	0.04

## Discussion

Carmustine (Cr) is an important chemotherapeutic drug for treating brain tumors, lymphoma, and multiple myeloma [23]. Genotoxicity is a common side effect of all chemotherapeutic agents which may lead to secondary malignancies. Herein the genotoxicity of Cr was evaluated using various cytogenetic parameters as the first goal of this work. To the best of our knowledge the present study is the first one referring to the previous works on Cr genotoxicity. The protective role of different doses of ethyl acetate fraction of *C. variegatum* leaves was also evaluated which represents the second goal of this study. Cr was found to induce a genotoxic effect in somatic cells evidenced by an increase in the frequency of MPCEs and CAs in bone marrow cells by about 3- and 7- fold increases over that of the negative control respectively. The percentage of the induced CAs in bone marrow reached 32.60 ± 0.75 compared with 4.60 ± 0.40 for the control. Cells with multiple aberrations were the most pronounced. This result is in the same line as that obtained in the study of Tates et al. [24]. The results of genotoxicity in bone marrow cells emphasize the positive correlation between micronucleus and chromosomal aberration induction. This indicates that one parameter has predictive value for the other. Fahmy et al. [25] suggested that CAs induced by chemical agents is essential events for the induction of MPCEs of bone marrow. The genotoxicity of Cr in bone marrow cells that is detected in the present work may be an indication for inability of this tissue to repair DNA damage induced by Cr efficiently [25, 26]. The results coincide with Tofilon et al. [27] who demonstrated that Cr induced sister chromatid exchanges in 9L rat brain tumor cells. The present results also demonstrated that Cr is toxic to bone marrow cells evidenced by increase in the percentage of PCEs/ (NCEs + PCEs). Previous studies recorded myelotoxicity and myelosuppression of bone marrow cells after Cr treatment [28]. El-Sayed et al. [29] reported that i.p injection of rats with Cr (30 mg/kg) significantly increased bone marrow apoptosis and bone marrow content of malondialdehyde which is a lipid peroxidation marker. Also, Lytle et al. [30] demonstrated that Cr chemotherapy down-regulates Bcl-xL and Bcl-2 in the bone marrow resulting in enhancement of apoptosis. Cr, like certain other nitrosoureas, decomposes spontaneously into 2 active intermediates: chloroethyl diazohydroxide and an isocyanate group. The DNA alkylation leads to the formation of irreversible DNA-DNA and DNA–protein crosslinks which are mediated by the chloroethyl diazohydroxide intermediate. The isocyanate intermediate produces carbamoylation of amino groups, which inhibits DNA repair, depletes glutathione and interferes with DNA/RNA synthesis [29].

Cr was reported to inhibit the antioxidant enzyme glutathione reductase (GR) massively [31]. As one of the most effective and abundant ROS-scavenging systems, glutathione plays a critical role in the maintenance of the redox balance in virtually all cells including normal and neoplastic cells. Therefore, inhibition of GR by Cr leads to the accumulation of reactive oxygen species which has been considered as one of the mechanisms responsible for Cr-genotoxicity and cytotoxicity. Recently, it was demonstrated that depletion of reduced GR enhanced the production ROS after opening the mitochondrial permeability transition pore [32]. ROS, such as the superoxide anion (O_2_^−^), hydrogen peroxide H_2_O_2_ and the hydroxyl radical, are thought to be involved in mediating the cytotoxic pathways induced by many anti-cancer agents [14, 33]. Cr also reduced the activity of other antioxidant enzymes SOD, CAT, and GPx [29]. It has been reported that GPx is capable of reducing free hydrogen peroxide, while CAT and SOD provide the first line defense against oxygen radical toxicity [34].

Concerning gonadal cells, the present results illustrated that Cr induced a significant percentage of CAs in mouse spermatocytes. The majority is in the form of univalent (X–Y and autosomal univalent). It also induced morphological sperm defects. These results are in the same line with the reports of Meistrich et al. [35].The univalent chromosome formation in spermatocytes is thought to be developed due to meiotic division that cannot be successfully completed. The study by Inanc et al. [23] finds a high level of caspase 3 in the cells undergoing meiosis after treatment with Cr. This could be explained the induction of univalent chromosomes and genotoxicity of Cr that ultimately reached to apoptosis in these cells as one of the defense mechanisms. Generally, it was reported that alkylating agents induce toxic effects on gonads [36]. Also some nitrosourea chemotherapeutic agents like 5-FU induced the same effect in mouse spermatocytes [14]. Cr in particular has been demonstrated to induce oxidative stress in the form of reactive oxygen species and lipid peroxidation in testicular tissues [37]. Lipid peroxidation has been reported to induce inflammation in gonads which may lead to toxic alterations in the cells associated with spermatogenesis and reduce spermatozoa motility. Cr generates ROS in testicular cells in the form of hydroxide radicals (OH), superoxide anions (O_2_^−^) and hydrogen peroxide H_2_O_2_ which can damage cellular macromolecules, DNA, RNA, protein and lipids in the testes [23].

It was recorded that the measurements of morphological sperm abnormalities could be used as an indication of damage that spermatogenic cells have suffered from chemical or physical agents [14]. The present results also recorded a significant percentage of morphological sperm abnormalities after Cr treatment with malformation of sperm head and tail. Coiled tail sperm was the most pronounced type of abnormalities induced after Cr treatment. The coiling of the sperm tail greatly affects its motility and function, accordingly this decrease its ability to fertilize an egg. These results are coincide well with the results of Inanc et al. [23] who reported that Cr negatively affected spermatozoa motility and induced sperm toxic effect as a result of oxidative stress and DNA damage. Defective sperm function has been identified as the most common cause of infertility [14].

The results of the present work also revealed an over expression of the gene p53 after Cr treatment which may be an indication of cellular DNA damage. Most chemotherapeutic drugs cause DNA damage, which is sensed by p53; the cell can then try to repair the damage or induce cell suicide [38].

Nowadays, the search for new and effective antioxidants is of a great importance especially, that extracted from plant origin. Herein the ethyl acetate fraction of *C. variegatum* leaves was evaluated as a positive chemo-preventive agent. Its genotoxicity/anti-genotoxicity was discussed and the main effective constituents were evaluated. The results showed that *C. variegatum* has no genotoxic effect in all tests examined. Moreover, a significant decrease in MPECs, CAs, and the toxicity of bone marrow in the combined groups treated with the plant and Cr was recorded in comparison with Cr group. The results also showed that *C. variegatum* could potentially protect against genotoxicity in germ cells. The results demonstrated about 50% decrease in the percentage of Cr- induced MPCEs, CAs in bone marrow and mouse spermatocytes with the highest tested dose of the extract. Also, a dose-dependent decrease was reported in sperm abnormalities in the combined groups (Cr and plant). *C. variegatum* is known to have medicinal properties like antioxidant, anti-inflammatory and anticancerious properties [2]. In a previous study, the phenolic compounds extracted from *C. variegatum* leaves showed an ameliorative effect against CAs induced by mitomycin C (MMC) in bone marrow and mouse spermatocytes. Likewise, the extract declined cytotoxicity and DNA damage (as indicated by comet assay) induced by MMC in bone marrow cells [39]. The same authors demonstrated that the highest concentration (500 mg/kg) of this extract did not induce cytotoxicity, CAs, or DNA damage. Moreover, it was reported that the aqueous extract from *C. variegatum* did not induce cytotoxic or genotoxic effects on mouse lymphoma cells [2, 39]. The goal of HPLC-*q*TOF-MS/MS analysis was performed to identify and quantify all secondary metabolites belonging to different chemical groups. The flavonoids (including flavones (35.21%) of *C*- and/or *O*-hexosides nature, flavonols (3.99%) and phenolic acids (17.62%), were the main identified polyphenols. Polyphenols have antioxidant activities and could improve sperm parameters. Plants extremely rich in natural antioxidants, such as flavonoids and phenolic acids have a protective role against genotoxic hazards both in vitro and in vivo [14]. Our study is characterized by a large range of *C*-glycosylflavonoids and vitexin was the main *C*-flavone glycoside (28.97%). Vitexin is a powerful antioxidant against ROS and lipid peroxidation. Vitexin activates the signaling pathways that depend on its antioxidant activity and has anti-inflammatory and antiangiogenic properties [40]. Isovitexin, orientin and their multiglycosides such as vicenin-2 were detected in the fraction. These compounds were also previously isolated from *C. variegatum* leaves [41]. Different biological properties including antioxidant, anti-cancerous, anti-inflammatory and chemo-preventive have been attributed to the *C*- and *O*-glycosyl flavones content of flavonoids [42]*.* Flavones are the most likely candidates among the compounds known for preventing oxidative lesions and providing antigenotoxic effects. Many other flavones were also identified such as rhoifolin, acacetin, apigenin, and diosmin with the amount range of 0.17–2.08%. The flavone; rhoifolin was reported to possesses a variety of significant biological activities including anti-inflammatory, antioxidant, and anticancer effects [43]. Acacetin is known to possess numerous pharmacological properties, including cardioprotective, neuroprotective, anti-inflammatory, antidiabetic and anticancer activities [44, 45]. Apigenin is known for its significant antioxidant and anti-proliferative activity and scavenges reactive oxygen species. Some derivatives of kaempferol were also identified (0.32–1.62%). This class of polyphenol secondary metabolites was demonstrated to have anti-proliferative activities on a variety of human cancer cell lines [40]. Phenolic acids (17.61%) were the second main group of compounds. Some phenolic acids showed an ameliorative effect on sperm DNA damage caused by oxidative stress and on testicular function and semen quality in human and animal models exposed to toxins. One of identified phenolic acids was rosmarinic acid (1.58%). Recently, the compound was reviewed in many in vitro and in vivo studies for its potent anti-inflammatory effects against inflammatory diseases [46]. In the current study different mono- and di-glycoside of quercetin were also identified (0.92%). The plant extract that contains quercetin and/or its derivatives has been reported to have antioxidant and antimutagenic properties [47]. In human lymphocytes, quercetin and some of its derivatives have shown a protective effect against oxidative DNA damage in vitro. Also, the anti-mutagenic activity against some oxidative mutagens in the Ames assay was reported [47]. Of the important compounds detected coumarins which represent 8.29% of the extract. Coumarin and its derivatives represent one of the most active classes of compounds that display a wide range of biological activities *e.g*. anti-inflammatory, antioxidants, anticancerious, radical scavenging activities as well as antibacterial and anti-HIV [48]. Indolic compounds (4.10%) are proven to be very efficient antioxidants, protecting cell proteins and lipids from peroxidation. The indole structure influences the antioxidant efficacy in biological systems [49].

In conclusion, the bioactivity of *C. variegatum* which was detected in the present work by inducing anti-genotoxicity in somatic and germ cells of male mice could be attributed to the major compounds or synergy between the major and minor constituents which can act by different mechanisms *e.g.* free radical scavengers, immune-stimulants, DNA repairs, termination of oxidative stress and activation of antioxidant enzymes.

## Data Availability

The datasets generated during and/or analysed during the current study are available from the corresponding author on reasonable request.
